# Optimizing the structure of urban woody plantings for a warming climate: a frequency-weighted exposure framework for woody species in Chongqing, China

**DOI:** 10.3389/fpls.2026.1889151

**Published:** 2026-08-21

**Authors:** Minxin Gou, Jie Xu, Xinyao Zhou, Haiyang Wang, Yangyang Sun

**Affiliations:** 1 College of Horticulture and Landscape Architecture, Southwest University, Chongqing, China; 2 College of Resources and Environment, Southwest University, Chongqing, China; 3 Interdisciplinary Research Center for Agriculture Green Development in Yangtze River Basin, Southwest University, Chongqing, China; 4 College of Landscape Architecture, Sichuan Agricultural University, Chengdu, China

**Keywords:** frequency-weighted exposure, optimization of use structure, plot-level occurrence frequency, thermal niche threshold, urban woody plants

## Abstract

As climate warming intensifies, woody plants in urban green-space systems are increasingly exposed to heat stress. Climate-adaptive assessment of urban woody species has often focused on species-level thermal tolerance, but system-level exposure also depends on how widely each species is used within existing planting structures. We used Chongqing, a major inland megacity in subtropical China, as a case study to demonstrate a frequency-weighted heat-stress exposure (HSE) framework for urban woody species. By integrating species-specific thermal niche thresholds with plot-level occurrence frequency, we developed a frequency-weighted HSE index to quantify the contribution of individual woody plant species to system-level heat exposure under future climatic conditions. Using survey data from 257 woody plant species across 310 plots in Chongqing, we found that HSE is highly concentrated among a subset of species in the current planting structure of the surveyed woody-plant system. The top 10 species with the highest HSE values accounted for nearly 30% of total system HSE, and the Herfindahl–Hirschman Index (HHI) of exposure concentration was 0.01525. To address this issue, we developed a strategy to optimize patterns of species use while maintaining the current proportions of species from various functional groups (evergreen trees, deciduous trees, etc.). Specifically, we reduced the occurrence frequency assigned to high-HSE species and analytically reallocated the released frequency to lower-deficit species from the same functional groups. Under the default optimization scenario, the optimized configuration reduced total system HSE by 2.02%, Top10_share by 3.26%, and HHI by 0.00254, demonstrating a reduction in reliance on a small number of commonly occurring high-HSE species. This study provides a quantitative basis for species selection, nursery stock procurement, and renewal strategies, supporting the climate-responsive management of woody-plant systems in urban green spaces.

## 1 Introduction

As the climate warms, woody plants in urban green-space systems will be subjected to greater heat stress (Li et al., 2025). Associated exposure at the system level will be determined not only by the characteristics of individual woody species but also by their plot-level occurrence frequency within urban planting structures (Bai et al., 2024; Zhu et al., 2025). For woody-plant systems in urban green spaces that rely on long-term species selection, planting renewal, and continuous service provision, assessment based on the ecological niches of individual species is important for evaluating their potential stress exposure under future climate conditions (Esperón-Rodríguez et al., 2021, 2024, 2025). At the same time, system-level exposure in urban green spaces depends not only on the future exposure of individual species to high temperatures but also on the breadth of their use in existing planting structures (Sun et al., 2026). When cities rely primarily on a small number of frequently occurring woody species, even if these species are not the most sensitive to high temperatures, their high occurrence frequency may still amplify the total heat-stress exposure (HSE) of the system and concentrate future exposure in a few commonly occurring high-HSE species (Werbin et al., 2020; Keenan et al., 2025). Such an exposure pattern may weaken the buffering capacity of community composition and increase the uncertainty surrounding management practices and the maintenance of landscape function (Iungman et al., 2023).

Recent research on climate risks to urban tree species has focused mainly on the identification of high-risk species on the basis of heat-tolerance thresholds, ecological niche boundaries, or future suitability changes (Gala et al., 2025; Kim et al., 2025; Zhu et al., 2025). Although such studies provide important information for the adaptation of urban green spaces to climate change, they focus mainly on exposure assessment at the species level and pay less attention to the structure of woody-plant assemblages at the systems level. In particular, the climate sensitivities of individual species are rarely combined with plot-level occurrence frequency, which can be used as a proxy for the breadth of species used in surveyed urban green-space woody-plant systems. We therefore lack clear quantitative information on whether current planting structures have concentrated future HSE in a few frequently occurring species and whether there is room for optimizing this exposure pattern without changing the current proportions of species from various functional groups.

**Figure 1:**
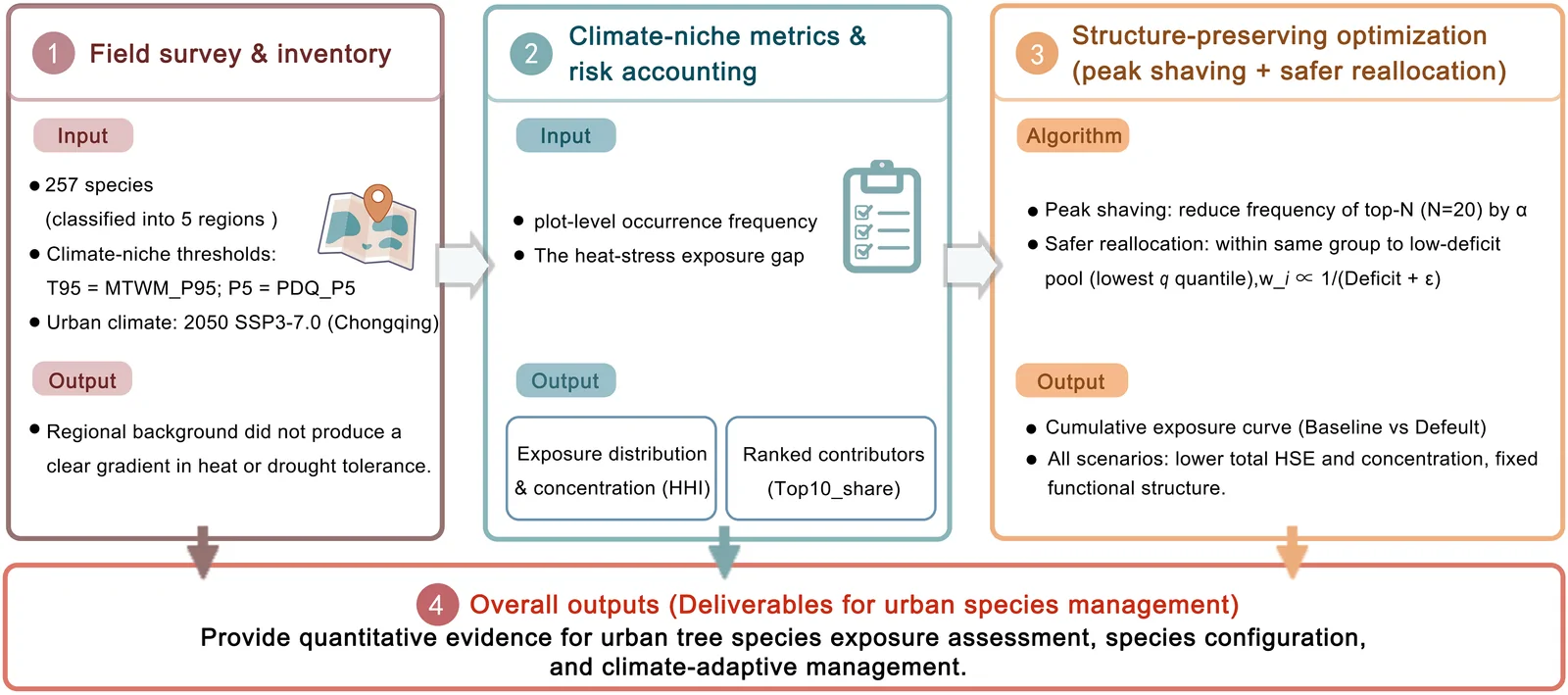
Workflow for structure-preserving optimization of frequency-weighted heat-stress exposure (HSE). Field survey data, climate-niche thresholds, and projected 2050 climate under Shared Socioeconomic Pathway 3-7.0 (SSP370) were combined to quantify HSE distribution and concentration. High-HSE species were then reduced through peak shaving, and the released frequency was reallocated to lower-deficit species within the same functional group, thereby lowering total HSE and HSE concentration while preserving functional structure. MTWM_P95, 95th percentile of the maximum temperature of the warmest month; PDQ_P5, 5th percentile of precipitation of the driest quarter; HHI, Herfindahl–Hirschman Index; Top10_share, share of total HSE contributed by the top 10 species.

With these issues in mind, we selected Chongqing, a subtropical inland megacity that has experienced recurrent heat waves, as the case-study city (Huang et al., 2022). By integrating woody-plant survey data, species-specific thermal niche thresholds, and information on future climate scenarios, we developed a frequency-weighted HSE metric based on the product of the HSE gap and the plot-level occurrence frequency. Compared with conventional species-level screening, this metric adds a structural dimension by considering not only each species’ projected heat-exposure gap but also how widely it occurs across the surveyed plots, thereby capturing its contribution to system-level exposure. Here, the HSE gap represents the degree to which projected future thermal conditions exceed a species’ thermal niche threshold; it is used to assess the distribution of heat-stress exposure at the system level under the current planting structure. On this basis, we propose an approach for optimizing species use while maintaining current functional-structure constraints. Specifically, although the frequency of each plant functional group is unchanged, the use frequency of high-HSE species is reduced, and the released frequency is analytically reallocated to species with lower HSE gaps within the same functional group. Because the patterns of woody plant use in the urban environment are shaped, to some extent, by species’ regions of origin, and because potential differences in thermal and drought niche thresholds among species from different origins may influence the distribution of system-level exposure, we aimed to address the following questions (**Figure 1**):

1. Do broad region-of-origin categories explain variation in the thermal and drought niche thresholds used as inputs to the HSE framework?
2. Under the current planting structure, how concentrated is frequency-weighted HSE among species, and how does frequency weighting alter species prioritization relative to Deficit_MTWM alone?
3. Under the constraint of maintaining functional-group structure, to what extent can optimization of species use reduce total system HSE and improve the distribution of exposure? Addressing these questions will help to extend heat-exposure assessment for urban woody species beyond species-level threshold identification toward the diagnosis and optimization of species-use structure at the system level.

## 2 Materials and methods

### 2.1 Study area, data sources, and extraction of climatic niche thresholds

The investigated woody vegetation consisted of urban woody-plant assemblages recorded in 310 sampling plots across the main urban districts of Chongqing. Each plot covered an area of 400 m². The plots were selected to cover the major types of urban green space in the city, and their distribution among green-space types is provided in **Supplementary Table 1**. Plot-level occurrence frequency in this study thus represents species use within the surveyed system, rather than a complete census of all woody plants or all urban green spaces in Chongqing. The study area and plot design, compilation of the species inventory, and procedures for data cleaning were described in our previous work (Xu et al., 2026). After data compilation, we obtained use-frequency data for 257 surveyed species in Chongqing. A functional-group summary of the species inventory, plot occurrence frequencies, Deficit_MTWM values, and HSE values is provided in **Supplementary Table 2**. The species were classified into five regions of origin according to the areal-type system of seed plant genera in China (Wu et al., 2003): cosmopolitan (8 species, 3.1%), tropical (124 species, 48.2%), northern temperate (89 species, 34.6%), paleo-Mediterranean (4 species, 1.6%), and east Asian (32 species, 12.5%). This classification enabled us to examine whether broad region-of-origin categories explained variation in the climatic niche thresholds used as inputs to the HSE framework. We selected maximum temperature of the warmest month (MTWM) and precipitation of the driest quarter (PDQ) as the key bioclimatic variables, referring to previous studies on climate-risk assessment of urban plants (Esperón-Rodríguez et al., 2024). Using historical climate data from WorldClim 2.1 (1970–2000) and future climate projections under the SSP370 scenario, we extracted climatic values corresponding to the distribution records of each species. The 95th percentile of MTWM and the 5th percentile of PDQ were then used to define the thermal niche threshold (T95) and drought niche threshold (P5) of each species. T95 and P5 were treated as point estimates of species-level climatic niche thresholds for subsequent HSE calculations. These percentile-based thresholds should be interpreted as approximate niche boundaries rather than direct physiological tolerance limits, because they may be influenced by the number of species occurrence records, spatial sampling bias in the distribution data, the resolution of climate layers, and the choice of percentile thresholds. PDQ was primarily used to compare differences in drought-niche thresholds among plants from different regions, whereas analyses of system HSE and structural optimization focused on future heat risk. The following analyses are therefore based mainly on MTWM.

### 2.2 Heat exposure gap and frequency-weighted exposure

The HSE gap (Deficit_MTWM) for each species was calculated on the basis of its thermal niche threshold (T95) and the projected MTWM value for the Chongqing study area under the SSP370 scenario in 2050:


Deficit_MTWM=max(0, MTWM_future−T95)

Thus, the HSE gap was positive only when the future MTWM exceeded the species’ thermal threshold; otherwise, it was set to 0. On this basis, the species-specific HSE gap was combined with its observed plot-level occurrence frequency across sample plots to construct a frequency-weighted exposure metric:


HSE=freq×Deficit_MTWM

where freq denotes the number of plots in which a given species occurred among the 310 sampling plots and is used as a proxy for the breadth of species use in the surveyed urban green-space woody-plant system, and Deficit_MTWM denotes the corresponding species-specific HSE gap. This exposure metric is then used to characterize the contribution of a given species to the total system-level HSE under the existing planting scheme. Frequency-weighted HSE values were calculated for all 257 species, and the sum of these values was taken to represent the total system exposure:


Total HSE=∑HSE_i

where HSE_*i* is the frequency-weighted HSE value of species *i.* On this basis, we analyzed the interspecific distribution of future HSE and its degree of concentration in individual species. Because all species were evaluated across the same 310 plots, normalization of occurrence frequency by the total number of plots would multiply all species-level HSE values and Total HSE by the same constant, without changing species rankings, Top10_share, HHI, or proportional comparisons among scenarios. We therefore retained plot counts for interpretability and treated absolute HSE as a system-specific index.

### 2.3 Total system HSE and concentration metrics

We used three indicators to characterize the distribution of future HSE under the current tree planting scheme in Chongqing: Total HSE, the HSE share of the top ten ranked species (Top10_share), and the Herfindahl–Hirschman Index (HHI).

Top10_share represents the proportion of total system HSE contributed by the 10 species with the highest HSE values and was used to reflect the degree to which the system depends on a small number of high-HSE species; higher values indicate a greater concentration of HSE in a few frequently occurring high-HSE species.

The Herfindahl–Hirschman Index (HHI) was used to characterize exposure concentration and was calculated as:


$$\text{HHI}=\sum {\left(\text{HSE}\_i/\text{Total HSE}\right)}^{2}$$

where HSE_*i*/Total HSE represents the exposure share of species *i*. A higher value of HHI indicates that system-level exposure is more concentrated in a small number of frequently occurring high-HSE species, whereas a lower value indicates a broader distribution of exposure across species. Together, these indicators were used to characterize the overall level and distribution pattern of system-level HSE under the baseline planting scheme.

To assess whether frequency weighting changed species prioritization, we ranked all 257 species separately by Deficit_MTWM and HSE. We compared the two rankings using Spearman’s rank correlation and quantified the overlap between their top-20 lists. Rank shift was calculated as the Deficit_MTWM rank minus the HSE rank; positive values indicate a higher rank under HSE. We used the top 20 because these species formed the donor set in the optimization analysis.

### 2.4 Testing for differences in niche thresholds among species from different regions

To test whether ecological niche thresholds differed significantly among species from different regions, we analyzed T95 and P5 separately as response variables, with region as the grouping variable. Given the unequal sample sizes among groups and the possibility that threshold distributions did not satisfy the assumption of normality, comparisons were performed using the Kruskal–Wallis rank-sum test. Where the omnibus test was performed, pairwise comparisons among region-of-origin categories were further examined using Dunn’s *post-hoc* tests with Holm adjustment for multiple comparisons. The magnitude of the overall group effect was quantified using epsilon-squared (ϵ^2^).

### 2.5 A “peak shaving + safe reallocation” strategy for optimization under functional-group constraints

We next sought to optimize the selection of tree species while maintaining the relative proportions of different functional groups within the planting scheme. Species were first classified into five functional groups: evergreen trees, deciduous trees, evergreen shrubs, deciduous shrubs, and climbing shrubs. Before and after optimization, the total frequency of each functional group was kept constant; that is, frequency adjustments were confined within groups, with no reallocation across functional groups. The optimization procedure consisted of two steps: peak shaving and safe reallocation. First, species were ranked in descending order according to their frequency-weighted HSE values, and the 20 species with the greatest HSE contributions were selected as the donor set (donors). Their frequencies were then reduced according to the update intensity, α:


freq′_i= freq_i× (1 − α)

The frequency share made available through this process was then reallocated to species with lower HSE gaps from the same functional group. The candidate pool was first identified at the species level using the *q*-quantile threshold of Deficit_MTWM and then intersected with the species set of each functional group. A smaller *q* indicated a stricter screening criterion and thus a narrower candidate pool, whereas a larger *q* created a broader candidate pool. Within each candidate pool, the available frequency share was reallocated using inverse weighting based on the HSE gap, such that species with smaller gaps received larger reallocated shares. To avoid division by zero, a very small constant, ϵ, was added to the weight calculation:


w_i∝1/(Deficit_MTWM,i+ ϵ)

After reallocation, the total frequency of each functional group was unchanged, ensuring that the functional structure of the overall planting scheme was preserved. To examine the effects of update intensity and candidate-pool strictness on optimization outcomes, we focused on three representative scenarios: (1) a conservative scenario, with α = 0.10 and *q* = 0.30; (2) a default scenario, with α = 0.20 and *q* = 0.20; and (3) an upper-bound scenario, with α = 0.40 and *q* = 0.10. We also performed a grid search over parameter combinations of α ∈ [0.10, 0.30] and *q* ∈ [0.10, 0.30] to further examine the trade-offs under different parameter settings. Changes in Total HSE, Top10_share, and HHI relative to the baseline were compared across all scenarios to evaluate shifts in overall system HSE and its degree of concentration. Because the reallocation rule preferentially assigned released frequency to species with lower Deficit_MTWM values, a reduction in Total HSE was expected. Therefore, the optimization procedure was not intended to test whether Total HSE could be reduced *per se*. Instead, it was used as a constrained scenario analysis to examine how different update intensities and candidate-pool strictness would affect exposure concentration, functional-structure preservation, and potential within-group donor–recipient adjustment pathways.

## 3 Results

### 3.1 Niche thresholds did not differ significantly among species from different regions

After classifying the 257 woody plant species according to their regions of origin, we compared their thermal niche thresholds (T95) and drought niche thresholds (P5). Kruskal–Wallis tests detected no statistically significant differences among regions of origin for either T95 (H = 2.193, df=4, *p* = 0.700, ϵ^2^<0.001) or P5 (H = 5.232, df=4, *p* = 0.264, ϵ^2^ = 0.004). Dunn’s *post hoc* tests with Holm adjustment likewise identified no statistically significant pairwise differences for either threshold (all adjusted *p*>0.05). Thus, for the species examined in this study, regional background explained little of the detectable variation in thermal or drought niche thresholds. These results suggest that, at least for the species examined here, historical distribution alone is insufficient to characterize differences in future heat risk among species. On this basis, we next characterized the distribution of system-level HSE from the perspective of species-use structure.

### 3.2 Frequency weighting reveals concentrated HSE and changes species prioritization

Among the 257 species with frequency data in Chongqing, all species had positive Deficit_MTWM values under the projected 2050 climate scenario (**Supplementary Table 2**). The baseline total system HSE was 8,298.98. Exposure contributions were concentrated in a subset of species: the top 10 species accounted for 29.92% of total HSE (Top10_share = 0.2992), and the HHI was 0.01525. These results indicate that, under the current planting scheme, system-level HSE is influenced primarily by a small number of frequently occurring high-HSE species. Among the top contributors, high HSE was associated with high plot occurrence frequency, large Deficit_MTWM, or a combination of both (**Supplementary Table 3**).

Rankings based on Deficit_MTWM and HSE were weakly correlated (Spearman’s ρ = 0.226, p < 0.001), and only one species, *Lagerstroemia indica*, appeared in both top-20 lists (5%; **Supplementary Table 4**; **Supplementary Figure 2**). Species with high occurrence frequency tended to rank higher under HSE, whereas species with large Deficit_MTWM but low occurrence frequency tended to rank lower. Thus, frequency weighting substantially changed which species were identified as the largest contributors to system-level exposure.

**Figure 2:**
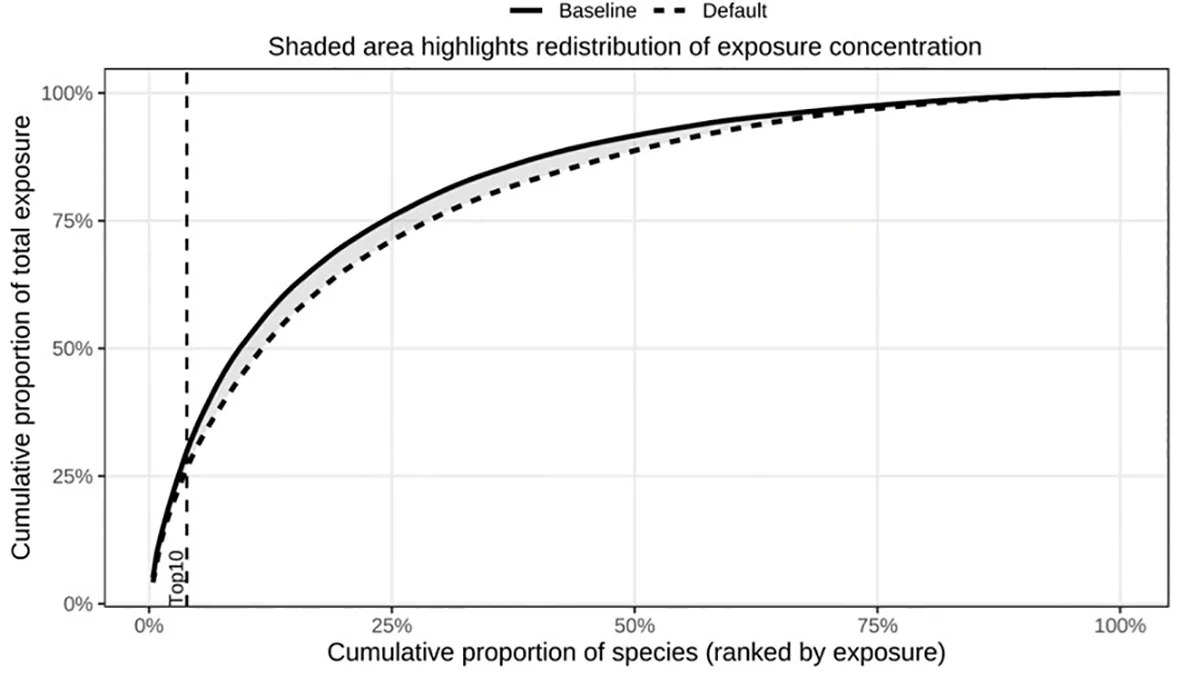
Cumulative exposure curve (baseline vs. default scenario).

The cumulative exposure curve further illustrates this concentration pattern (**Figure 2**). Under the baseline scheme, the cumulative curve, ranked by species’ HSE contributions, initially rises steeply, indicating that total exposure accumulates rapidly among a small number of highly exposed species. By contrast, under the default optimization scenario, the initial portion of the cumulative curve lies consistently below that of the baseline, suggesting that the exposure contributions of the frequently occurring high-HSE species have declined and that exposure is more evenly distributed across species. Under the default scenario, Top10_share decreased from 0.2992 to 0.2666.

### 3.3 Structure-preserving optimization redistributes exposure under functional-group constraints

As expected from the reallocation rule, all three main optimization scenarios (conservative, default, and upper bound) reduced the total HSE. More importantly, these scenarios also reduced exposure concentration while strictly maintaining the functional structure of the planting scheme. For each plant functional group, the difference in total frequency before and after optimization was zero, indicating that optimization did not alter functional-group composition (**Table 1**).

**Table 1:** Effects of the three main-text optimization scenarios (top 20, functional structures maintained).

Scenario	α	*q*	removed_share	TotalHSE_change	Top10_change	HHI_change
Conservative	0.1	0.3	4.53%	−0.92%	−2.32%	−0.18%
Default	0.2	0.2	9.06%	−2.02%	−3.26%	−0.25%
UpperBound	0.4	0.1	18.12%	−8.49%	−8.87%	−0.51%

Under the conservative scenario, the adjusted frequency share was 0.0453, meaning that 4.53% of the occurrence frequency was shifted to species with a lower HSE value. Relative to the baseline scenario, the conservative scenario reduced total HSE by 0.92% (ΔTotal HSE = −0.00915), Top10_share by 2.32%, and HHI by 0.175%.

Under the default scenario, the adjusted frequency share increased to 0.0906. Total HSE was reduced by 2.02% (ΔTotal HSE = −0.02017), Top10_share by 3.2%, and HHI by 0.25%. In **Figure 2**, the cumulative exposure curve under the default scenario is shifted downward relative to the baseline across its entire trajectory, highlighting the reduction in HSE contributions of frequently occurring high-HSE species.

Under the upper-bound scenario, the adjusted frequency share reached 0.1813, and the optimization effect was most pronounced. In this scenario, total HSE was reduced by 8.49% (ΔTotal HSE = −0.08491), Top10_share by 8.9%, and HHI by 0.51%.

**Figure 3:**
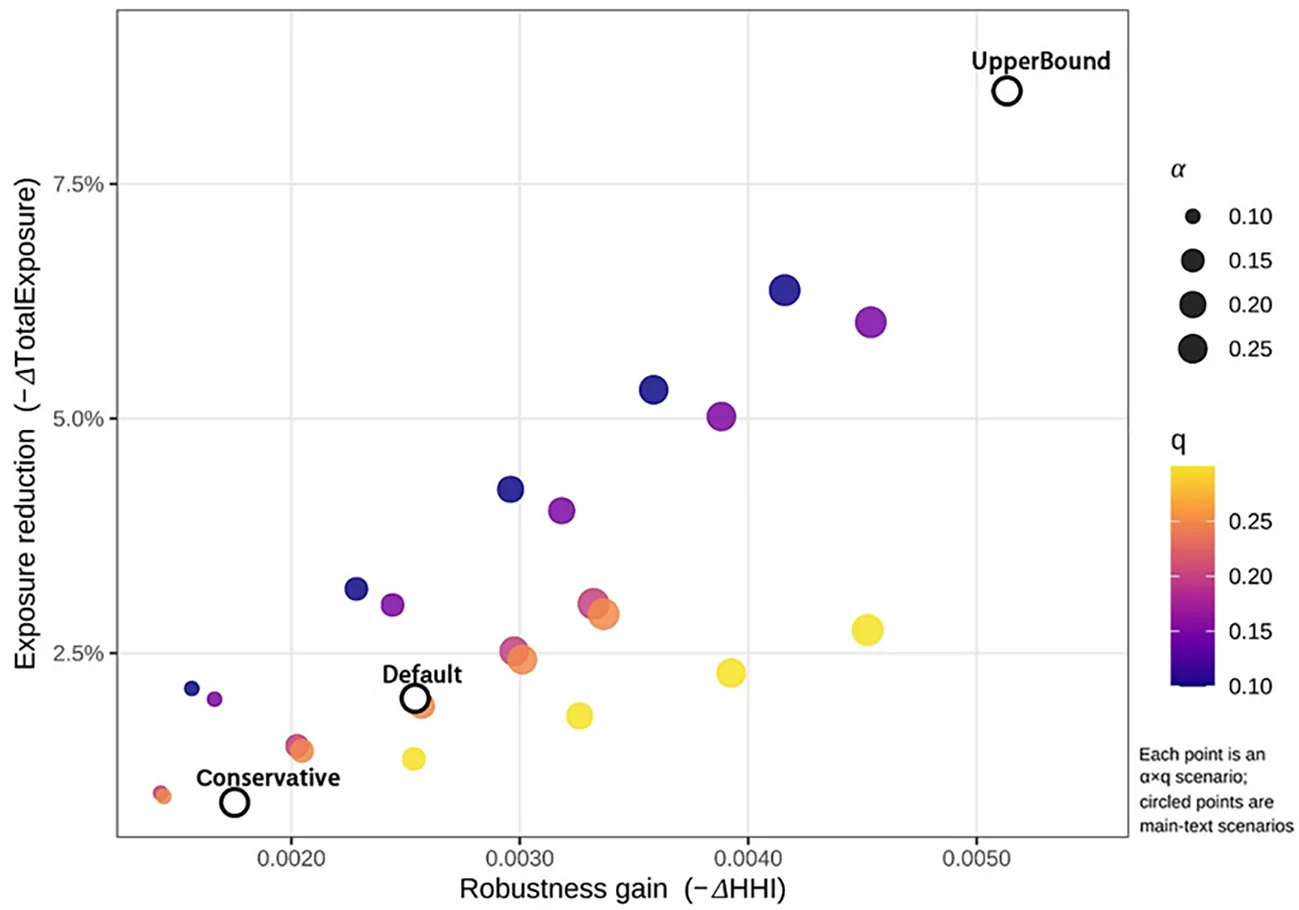
Scatter plot of α×*q* trade-off (with the functional structure of the planting scheme preserved).

**Figure 3** illustrates the trade-offs under different α × *q* parameter combinations. The corresponding sensitivity of Total HSE and HHI reductions across the α × q parameter grid is shown in Supplementary **Figure 1**. Overall, as α increased, the reductions in total system HSE and HSE concentration became more pronounced. At higher levels of α, lower values of *q*—that is, stricter screening of the low-deficit candidate pool—generally produced stronger optimization effects. The three main scenarios highlighted in the figure (conservative, default, and upper-bound) represent a gradient from weaker to stronger intervention, showing how greater adjustment intensity and stricter candidate selection can redistribute exposure away from a small subset of high-HSE species while preserving functional-group structure.

## 4 Discussion

Within the urban woody-plant system of Chongqing, neither the thermal niche threshold (T95) nor the drought niche threshold (P5) differed significantly among species from different regions of origin. Thus, for the species examined here and at the present level of classification, historical regional background is not associated with clear differences in climatic threshold and cannot be used, on its own, to predict future patterns of system-level heat-stress exposure. By contrast, system-level HSE is more directly linked to the HSE gaps of individual species and to patterns of species use. When certain species are used extensively in urban green spaces, the effects of their HSE gaps are amplified by the planting structure, increasing both Total HSE and the concentration of that exposure. From the perspective of urban woody-plant management, greater attention should therefore be paid to the extent to which species with positive heat-exposure deficits are currently used in the surveyed planting system and whether such use has already resulted in excessive dependence on a small number of frequently occurring species (Bingqian et al., 2020; Hilbert et al., 2023).

Our findings also indicate that, in management practice, merely knowing which species have higher heat-exposure deficits is not sufficient; it is also necessary to determine the frequency and exposure contribution of these species within the existing planting structure (Feng et al., 2022; Harper et al., 2022). Woody-plant systems in urban green spaces are not formed by the simple aggregation of independent species; rather, they embody long-established configuration preferences and path-dependent patterns of use (Romero-Muñoz et al., 2024). The weak correspondence between the Deficit_MTWM and HSE rankings illustrates the value of integrating species-level heat-exposure gaps with plot-level occurrence frequency. Deficit_MTWM identifies species with the largest projected heat-exposure gaps, whereas HSE identifies those contributing most to exposure under the current planting structure. Accordingly, species-level HSE should be interpreted as a within-system indicator of exposure contribution rather than as a stand-alone management threshold. Species with large Deficit_MTWM but moderate occurrence frequency may warrant monitoring and cautious future use, whereas species with moderate deficits but high occurrence frequency may represent priorities for gradual replacement; both components should therefore be considered separately alongside HSE.

Against this background, the peak shaving + safe reallocation strategy proposed here can be seen as a constrained scenario-based approach for exploring gradual species-use optimization. In urban green-space renewal, adjustments to species configuration are typically constrained by multiple factors, including landscape functions, nursery stock availability, maintenance capacity, and pre-existing vegetation structure; accordingly, changes in planting configuration can rarely be achieved through one-time, large-scale replacement (Liu and Russo, 2021; Lu et al., 2024). The optimization framework developed in this study places particular emphasis on reducing the occurrence frequency of commonly used high-HSE species and analytical reallocation of the released frequency to lower-deficit species from the same functional group, thus providing a feasible strategy for adjustment under functional-structure constraints. Because HSE is calculated as the product of plot-level occurrence frequency and Deficit_MTWM, this reallocation rule is expected to reduce Total HSE. Therefore, the added value of the optimization procedure lies not simply in showing that Total HSE can be reduced, but in examining how exposure concentration and dependence on a small number of high-HSE species can be reduced while maintaining the broad functional-group structure of urban plantings. Under the default scenario, Total HSE showed a modest relative reduction of 2.02%. Although the magnitude of this reduction is not large in itself, the concurrent changes in Top10_share and HHI are more noteworthy. Both indicators declined with optimization, indicating that the effects of adjustment are reflected not only in a reduction in Total HSE but also in a reorganization of HSE distribution. As the exposure share borne by a few commonly occurring high-HSE species was reduced, the system’s dependence on these species was correspondingly weakened. This shift has direct structural implications for woody-plant systems in urban green spaces. Heat exposure is likely to be concentrated in a small number of frequently occurring species that make large contributions to system-level HSE. When these species become less stable under future climate scenarios, ecosystem service provision, landscape continuity, and maintenance pressure may all be affected simultaneously (Wood and Dupras, 2021; Wessely et al., 2024). Therefore, reducing the concentration of HSE is ecologically meaningful not simply because it lowers the total exposure value, but because it redistributes exposure away from a narrow set of commonly used high-HSE species. From a long-term management perspective, adjustments of this kind—small in magnitude but clear in direction—are more readily incorporated into annual renewal, nursery stock procurement, and phased optimization processes and are therefore more likely to be sustained over time.

This study still has several limitations. First, the extent to which MTWM exceeds species-specific thresholds was used to characterize potential heat-stress exposure. This metric reflects the potential exposure to heat stress under future warming scenarios rather than actual mortality rates or the probability of species decline (Xu et al., 2021; Gou et al., 2025). Factors such as the spatial heterogeneity of the urban heat island, soil conditions, irrigation management, pests and diseases, and local microenvironmental conditions may all influence the actual responses of plants to heat stress (Cao et al., 2024; Sun et al., 2025). Therefore, the results of this study are more appropriately interpreted as a screening-level basis for structural optimization and exposure assessment. In addition, T95 and P5 were estimated from species distribution records and should be interpreted as realized climatic niche thresholds rather than direct physiological tolerance limits. They may reflect not only species climatic tolerances, but also dispersal history, biotic interactions, human cultivation, management practices, and sampling bias in occurrence records. Uncertainty in the estimation of T95 and P5 was not explicitly quantified in this study. Such uncertainty may propagate into Deficit_MTWM and subsequently influence species-level HSE and system-level exposure metrics. Future studies could assess the robustness of these estimates by using bootstrap resampling of species occurrence records, controlled physiological tolerance data where available, multiple climate models and emission scenarios, and formal uncertainty propagation from niche-threshold estimation to HSE calculation.

Second, this study uses plot-level occurrence frequency to represent the breadth of species use in the surveyed urban green-space woody-plant system. Although this metric can effectively reflect how widely a species occurs across the surveyed plots, it is not equivalent to stem abundance, biomass, canopy cover, or ecosystem service contribution. Accordingly, the absolute value of Total HSE should be interpreted as a system-specific exposure index for comparing the baseline and optimization scenarios within the present survey system, rather than as an absolute risk value that can be directly compared across cities or sampling designs. Similarly, frequency changes under the optimization scenarios should be understood as analytical adjustments in species-use structure, rather than as direct changes in tree numbers, planting positions, or management replacement operations. For cross-city or cross-system applications, relative occurrence frequency or exposure-share-based metrics such as Top10_share and HHI should be used to reduce scale dependence. By incorporating stem abundance, biomass, canopy cover, and ecosystem service contribution, future studies could extend the exposure metric to abundance-weighted, canopy-weighted, or service-weighted forms of HSE.

Finally, this study is based on a single scenario (SSP370 for 2050) and a single urban environment. Different emission pathways, climate models, and urban contexts may all influence the pattern of exposure and the magnitude of optimization outcomes (Kacprzak et al., 2024; Zhu et al., 2025). Future research should therefore test the robustness and transferability of this framework across multiple climate scenarios and urban contexts. Overall, this study combines HSE assessment, plot-level occurrence frequency, and structure-preserving optimization into an integrated framework, providing a transparent analytical approach for exploring climate-adaptive adjustments in species use for woody-plant systems in urban green spaces. Its significance lies in helping to identify high-contributing species and potential for within-group adjustment that can be considered together with local management objectives, nursery stock availability, monitoring data, and phased green-space renewal.

## 5 Conclusion

We developed a frequency-weighted heat-stress exposure framework that links species-level thermal niche deficits with plot-level occurrence frequency in an urban woody-plant system. Broad region-of-origin categories did not reveal a clear gradient in thermal or drought niche thresholds, whereas future system-level heat exposure was more closely associated with the combination of species-specific heat-exposure deficits and species use structure. By reducing the use of high-HSE species and reallocating the released frequency to lower-deficit species within the same functional groups, the proposed optimization approach reduced exposure concentration while maintaining the broad functional structure of urban plantings.

Although the reduction in Total HSE under the default scenario was modest, the concurrent decrease in exposure concentration suggests that gradual within-group adjustment can reduce reliance on a small number of high-HSE species without altering the broad functional structure of plantings. The proposed framework should therefore be interpreted as a decision-support tool for identifying priority species and guiding phased species selection, nursery stock procurement, and green-space renewal, rather than as a direct prediction of tree mortality, canopy loss, or ecosystem service decline.

## Data Availability

The original contributions presented in the study are included in the article; further inquiries can be directed to the corresponding author.
