# Supplementary material for: Optimizing the structure of urban woody plantings for a warming climate: a frequency-weighted exposure framework for woody species in Chongqing, China
Source: Front Plant Sci. 2026 Aug 21;17:1889151. doi: 10.3389/fpls.2026.1889151 (PMC13541432; doi:10.3389/fpls.2026.1889151)
Supplement: Supplementary file 1 [file DataSheet1.pdf]

Supplementary information

Content of the supplementary information

Supplementary Figures 2

Supplementary Tables 4

Supplementary Figure

Figure S1. Sensitivity of Total HSE and HHI reductions across α × q combinations.

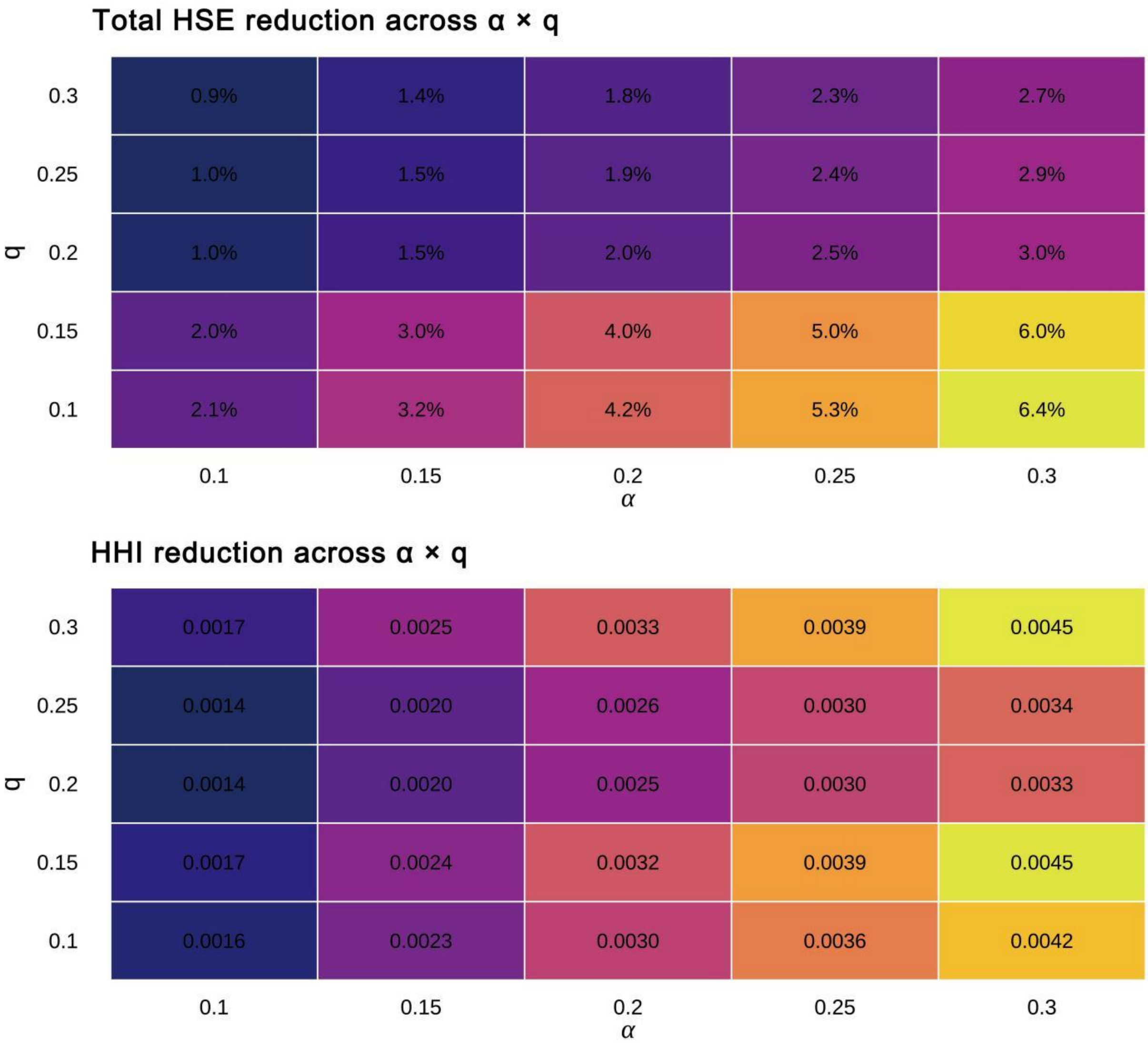

Figure S2. Comparison of species rankings based on Deficit_MTWM and frequency-weighted heat-stress exposure (HSE).

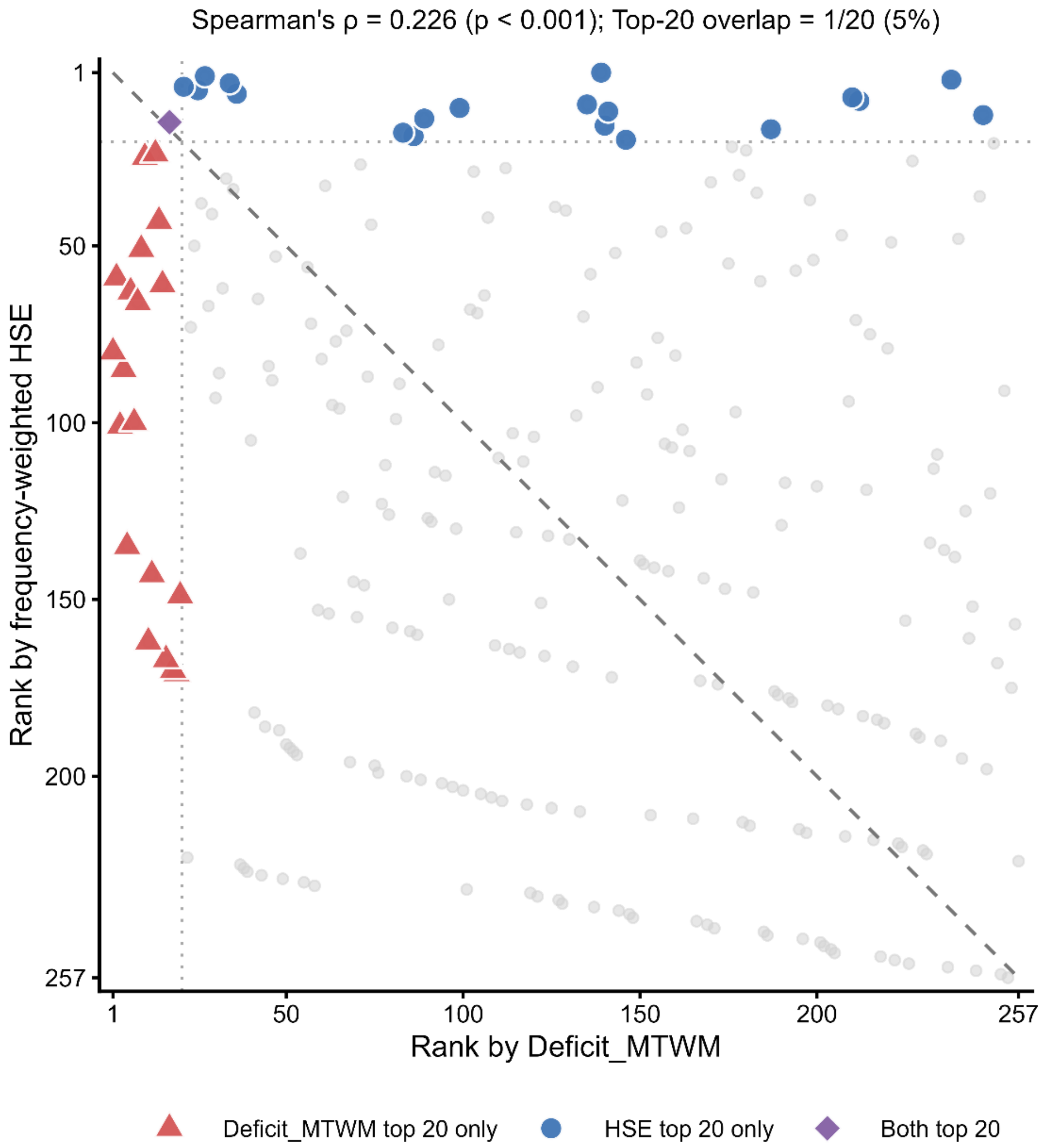

Supplementary Tables

Table S1. Distribution of sampling plots across different types of green spaces in main urban districts of Chongqing.

Type of urban green space	Name of site	Number of sampling plots
Pocket park	Shengjing Landscape Garden, Fengtian Ecological Park, Zourong Park, Bamboo Forest Park, Fenghuang Mountain Cultural Park.	16
Comprehensive park	Baiyun Mountain Park, Longtousi Park, Yubei, Jinshan Park, Futuguan Park, Pingdingshan Cultural Park, Chongqing Central Park, Geling Park, Garden Expo Park, Hutouyan Park, Jiangbeizui Central Park, University Town Central Park	79
Forest park	Zhaomu Mountain Forest Park, Hong'en Temple Forest Park, Jinyu Country Park	81
Themed park	Yudai Mountain Hillside Park, Jiuqu River Wetland Park, Golden Bay Park, Baiheju Ecological Garden, Tashan Park, Bijin Park, Botanical Garden	36
Community park	Baima Community Park, Xiaoxiajiawan Park, Taoyuan Park, Hong'en Temple Community Park, Shimen Park, People's Park	15
Protective green belt	Jialing Riverside Road, Shangtuwan Road, South District Road	13
District green space	Hongya Stockade Area, Liudiao Stream Area, Qingshui Stream Area, Hengliang Village, Wangjiaping New Village, Hongyan Village, Dashiba Village, Baimamiao Area, Beiheng Park Area, Baiyun Stockade Area, Fenghuang Terrace Area	40
Affiliated green space	Fenghuang Bay Area, Wanghai Flower Market Hillside, North Ring Interchange, East Ring Interchange, Jin'an Road, Shabin Road, Liziba Area	22
Public square	Caixin Plaza, Juxian Rock Plaza	8

Table S2. Summary of species inventory, plot occurrence frequency, projected heat-exposure deficit, and frequency-weighted HSE for the 257 woody plant species.

Functional group	All woody species	Evergreen trees	Deciduous trees	Evergreen shrubs	Deciduous shrubs	Climbing shrubs
Number of species	257	58	94	57	35	13
Species with Deficit_MTWM > 0, n (%)	257 (100.0%)	58 (100.0%)	94 (100.0%)	57 (100.0%)	35 (100.0%)	13 (100.0%)
Total plot occurrences	3674	600	1467	515	535	557
Plot occurrence frequency, median (IQR)	5.0 (2.0–14.0)	5.5 (2.0–11.8)	5.5 (3.0–15.0)	4.0 (2.0–8.0)	3.0 (2.0–14.0)	19.0 (10.0–53.0)
Deficit_MTWM, median (IQR), °C	2.146 (1.912–2.506)	2.157 (1.885–2.443)	2.173 (1.913–2.563)	2.146 (1.900–2.464)	2.120 (1.899–2.579)	2.082 (2.018–2.538)
Total HSE	8298.982	1285.464	3274.064	1227.974	1277.936	1233.545
Contribution to Total HSE (%)	100.00	15.49	39.45	14.80	15.40	14.86

Table S3. Top 20 species contributing to baseline frequency-weighted heat-stress exposure and decomposition of HSE into plot occurrence frequency and Deficit_MTWM.

**Species**	**Plot occurrence frequency**	**Deficit_MTWM (°C)**	**HSE**	**Contribution to total HSE (%)**
Broussonetia papyrifera	204	2.081999588	424.727916	5.12
Cinnamomum camphora	133	2.951599884	392.5627846	4.73
Ficus virens	152	1.73400116	263.5681763	3.18
Melia azedarach	83	2.817798233	233.8772533	2.82
Celtis sinensis	71	3.097999191	219.9579426	2.65
Loropetalum chinense var. *rubrum*	67	3.015200043	202.0184029	2.43
Erythrina variegata	71	2.812400818	199.6804581	2.41
Alangium chinense	103	1.848799133	190.4263107	2.29
Osmanthus fragrans	97	1.839000893	178.3830866	2.15
Koelreuteria bipinnata	84	2.113199425	177.5087517	2.14
Rhododendron simsii	71	2.291199875	162.6751911	1.96
Robinia pseudoacacia	70	2.080799484	145.6559639	1.76
Rhapis excelsa	90	1.617599487	145.5839539	1.75
Eriobotrya japonica	56	2.324799919	130.1887955	1.57
Lagerstroemia indica	40	3.210200882	128.4080353	1.55
Nandina domestica	59	2.081199455	122.7907679	1.48
Morus alba	64	1.914399719	122.521582	1.48
Ficus concinna	49	2.365001678	115.8850822	1.40
Ligustrum lucidum	48	2.342598915	112.4447479	1.35
Bischofia javanica	53	2.051998138	108.7559013	1.31

Table S4. Comparison of species rankings based on Deficit_MTWM alone and frequency-weighted heat-stress exposure (HSE) among species included in either top-20 list.

Species	Plot occurrence frequency	Deficit_MTWM (°C)	Rank by Deficit_MTWM	HSE	Rank by HSE	Rank shift (Deficit_MTWM rank − HSE rank)	Top-20 category
Broussonetia papyrifera	204	2.082	139	424.728	1	138	HSE top 20 only
Hedera nepalensis var. sinensis	4	6.540	1	26.160	80	-79	Deficit_MTWM top 20 only
Cinnamomum camphora	133	2.952	27	392.563	2	25	HSE top 20 only
Albizia kalkora	6	6.187	2	37.123	59	-57	Deficit_MTWM top 20 only
Ficus virens	152	1.734	238	263.568	3	235	HSE top 20 only
Cupressus funebris	3	6.156	3	18.467	101	-98	Deficit_MTWM top 20 only
Melia azedarach	83	2.818	34	233.877	4	30	HSE top 20 only
Populus canadensis	4	5.947	4	23.790	85	-81	Deficit_MTWM top 20 only
Celtis sinensis	71	3.098	21	219.958	5	16	HSE top 20 only
Prunus armeniaca	2	5.257	5	10.514	135	-130	Deficit_MTWM top 20 only
Loropetalum chinense var. *rubrum*	67	3.015	25	202.018	6	19	HSE top 20 only
Bauhinia blakeana	7	4.902	6	34.315	63	-57	Deficit_MTWM top 20 only
Erythrina variegata	71	2.812	36	199.680	7	29	HSE top 20 only
Rubus parvifolius	5	3.698	7	18.492	100	-93	Deficit_MTWM top 20 only
Alangium chinense	103	1.849	210	190.426	8	202	HSE top 20 only
Photinia serratifolia	9	3.632	8	32.688	66	-58	Deficit_MTWM top 20 only
Osmanthus fragrans	97	1.839	212	178.383	9	203	HSE top 20 only
Metasequoia glyptostroboides	12	3.522	9	42.262	51	-42	Deficit_MTWM top 20 only

Koelreuteria bipinnata	84	2.113	135	177.509	10	125	HSE top 20 only
Boehmeria nivea	23	3.442	10	79.157	25	-15	Deficit_MTWM top 20 only
Rhododendron simsii	71	2.291	99	162.675	11	88	HSE top 20 only
Houpoea officinalis	2	3.380	11	6.760	162	-151	Deficit_MTWM top 20 only
Robinia pseudoacacia	70	2.081	141	145.656	12	129	HSE top 20 only
Eucommia ulmoides	3	3.330	12	9.990	143	-131	Deficit_MTWM top 20 only
Rhapis excelsa	90	1.618	247	145.584	13	234	HSE top 20 only
Triadica sebifera	27	3.262	13	88.063	24	-11	Deficit_MTWM top 20 only
Eriobotrya japonica	56	2.325	89	130.189	14	75	HSE top 20 only
Solanum pseudocapsicum	16	3.235	14	51.760	43	-29	Deficit_MTWM top 20 only
Lagerstroemia indica	40	3.210	17	128.408	15	2	Both top 20
Ligustrum quihoui	11	3.221	15	35.431	61	-46	Deficit_MTWM top 20 only
Nandina domestica	59	2.081	140	122.791	16	124	HSE top 20 only
Paeonia delavayi	2	3.216	16	6.432	167	-151	Deficit_MTWM top 20 only
Morus alba	64	1.914	187	122.522	17	170	HSE top 20 only
Ficus concinna	49	2.365	83	115.885	18	65	HSE top 20 only
Viburnum odoratissimum	2	3.197	18	6.394	170	-152	Deficit_MTWM top 20 only
Ligustrum lucidum	48	2.343	86	112.445	19	67	HSE top 20 only
Senna corymbosa	2	3.183	19	6.366	171	-152	Deficit_MTWM top 20 only
Bischofia javanica	53	2.052	146	108.756	20	126	HSE top 20 only
Excoecaria cochinchinensis	3	3.164	20	9.492	149	-129	Deficit_MTWM top 20 only
